# Special Issue Editorial: Isolation and Analysis of Characteristic Compounds from Herbal and Plant Extracts

**DOI:** 10.3390/plants10122775

**Published:** 2021-12-15

**Authors:** Jong-Seong Kang, Narendra Singh Yadav

**Affiliations:** 1 College of Pharmacy, Chungnam National University, Daejeon 34134, Korea; 2 Department of Biological Sciences, University of Lethbridge, Lethbridge, AB T1K 3M4, Canada

Herbal and plant extracts exhibit various types of properties and activities that have been applied in the medicinal field to treat diseases and achieve better health. By isolating and characterizing the compounds in extracts, it helps to elucidate the pharmacological activities from the extract and explains the biochemical metabolism of compounds inside plants and herbs. In this Special Issue, thirteen research articles [1,2,3,4,5,6,7,8,9,10,11,12,13] and two reviews [14,15] focusing on isolation and analysis of compounds from plant extracts have been published. This Special Issue of *Plants* covers a broad range of recent findings of isolation, profiling, analysis of compounds from herbal and plant extracts, quality control, and standardization of processing extracts with characterized compounds.

The root from *Polygala tenuifolia* used in Asia is known to exhibit anti-inflammatory effects, which is used to treat asthma, bronchitis, and whooping cough. Vinh et al. isolated 15 compounds from methanolic extracts of *P. tenuifolia* root to discover new anti-inflammatory agents. As a result, they suggested potential candidates for the treatment of inflammation [1].

The dried leaves of *Istia indigotica* is well known for its anti-oxidative and anti-inflammatory effects. With those pharmacological activities, Folium isatidis showed superior anti-wrinkle effects compared to other herb and plants. Gao et al. analyzed antiwrinkle effect of extract and found an enriched fraction showing excellent anti-wrinkle effect. They identified 3,4,5-trimethoxycinnamic acid (TMCA) as a potential anti-wrinkle agent. Additionally, they developed analytical method of TMCA from Folium isatidis, which could be applied for quality control [2].

Cardiovascular diseases are disorders of heart and blood vessels including heart attack, hypertensive heart disease, and heart failure. In order to develop cardioprotective compounds from herb, Adhikari et al. investigated vasorelaxant effects from rhizomes of *Boesenbergia rotunda*. The methanol extracts from *B. rotunda* showed vasorelaxation effects and additional studies suggested that vasorelaxation effects are induced by relaxation in the coronary artery rings. Furthermore, a chromatographic study suggested that vasorelaxant effects are from flavonoids compound [3].

Oxidative stress could induce imbalance of free radicals and antioxidants, which could damage the human body’s cell, protein, and DNA. Tanruean et al. investigated antioxidant and biological activities of three Clauseneae plants: *Clausena excavate*, *C. harmandiana*, and *Murray koenigii*. Chemical properties of these plants were also evaluated regarding total phenolic and total flavonoids contents and essential oils. Together, this study suggested the extracts of three Clauseneae plants as potential candidate for natural bioactive agents [4].

Avocado oil has been reported to improve sensorineural hearing loss (SNHL). In order to identify the compound that improves SNHL, Park et al. isolated 20 compounds from avocado oil using column chromatography. Improvement of SNHL from the isolated compounds were evaluated using the ototoxicity zebrafish model. Among 20 isolated compounds, seven compounds showed significant improvement of SNHL. This studied confirmed that compounds from avocado oil have protective effect on damaged otic hair cells [5].

The fruit of *Schisandra chinenesis* (Omija) is well known for several biological activities from dibenzocyclooctadiene lignans. Park et al. compared the content of seven bioactive lignans from stem, leaf, pulp, seed, and flower of *S. chinensis* using HPLC-DAD. Additionally, contents of lignan in fermented beverages of *S. chinensis* were compared with different periods and different sugar composition. As a result, contents of lignan were the highest in seed, followed by fruit, flower, leaf, pup, and stem. Lignan content increased proportionally to fermentation period, and the Omija beverage fermented with oligosaccharide/white sugar had the highest contents of lignan compared to beverages fermented with other sugars [6].

Tepals of saffron (*Crocus sativus*) is known for its abundant bio-residues that could exhibit various bioactivities. Specifically, phenolic compounds and anthocyanins have been detected from saffron with antioxidant properties. Stelluti et al. compared extraction method of dried saffron tepals by comparing phytochemical composition. Collectively, content of phenolic compound from ultrasound assisted extraction method with safer solvent were comparable to the maceration method, which refers to the potential application of green extraction method for obtaining high yields of antioxidant agents [7].

Sarcoma is a malignant tumor originating from mesenchymal cells. Fibrosarcoma is malignant neoplasm that takes place in fibroblast. Proteases are involved in various biological processes, including cell cycle progression, cell adhesion, proliferation, and migration, which facilitates tumor progression. Im et al. evaluated plant-derived protease inhibitors to discover potential agents for cancer prevention. Several protease inhibitors were derived from seeds of *Bauhinia bauhiniodes* and *Enterolobium contortisiliquum*. The effect of these protease inhibitors was investigated on the mouse fibrosarcoma regarding cell viability, cell cycle, and cell adhesion. Collectively, with the positive outcome with effect of protease inhibitor, further studies including cell signaling and in vivo study will be needed for comprehensive understanding [8].

A fruit of Sea buckthorn (*Hippophae rhamnoides*) is well known for its nutrient composition, which is widely used. Lee et al. evaluated the phytochemical composition of the fruits of sea buckthorn to discover bioactive compounds. They isolated one malate derivative, five citrate derivatives, and one quinate derivative. The isolated compounds were analyzed by 1D and 2D nuclear magnetic resonance and high-resolution liquid chromatography-mass spectrometry (LC-MS) for structure identification. These compounds were evaluated for stimulatory effects on osteogenesis. Collectively, five compounds stimulated osteogenic differentiation, which can induce osteogenesis of mesenchymal stem cells [9].

The Dendrobium species has been used as herbal medicine for treatment of various disorders. Nam et al. investigated chemical profiles of Dendrobium in two different species, and hybrid and gamma-irradiated mutant lines were investigated with LC-MS. As a result, 17 compounds were identified from the Dendrobium, and the putative markers that contributed to the discrimination of active and inactive mutant lines were identified. Collectively, this study would be helpful for future investigations of mutation mechanisms and quality evaluation for the mutants [10].

1-Deoxynojirimycin (1-DNJ) is one of most widely used iminosugars from mulberry (*Morus* sp.pl.). This compound has been known to inhibit tumor cell metastasis and reduce fat accumulation. These pharmacological effects resulted in the wide use of mulberry leaves, which had the highest content during harvest season. Marchetti et al. investigated the influence of cultivation area and cultivars toward amount of 1-DNJ in leaves extracts using HPLC-MS. They found a comparable amount of 1-DNJ to Asian cultivations and high amount of 1-DNJ in summer and from apical leaves. Since many aspects affect the level of 1-DNJ, further study is needed for the standardization of 1-DNJ in mulberry leaves [11].

Inotodiol is well known for its wide range of pharmacological activities, which is from chaga mushroom, *Inonotus obliquus*. For further pharmacokinetic studies, development of determination method of inotodiol in biological matrix was necessary. Kim et al. developed novel bioanalytical method for the determination of inotodiol using LC-MS/MS. The method was successfully validated and applied to a pharmacokinetic study using mice model. The developed method would be further applied for the mechanistic study of inotodiol [12].

Flowers from Coreopsis have been used for ethnopharmacological purposes due to its known biological activities including anticancer, antioxidant, anti-inflammatory, and anti-diabetic effects. Kim et al. performed metabolic profiling and their inhibitory effect on dipeptidyl peptidase from five original cultivars and mutant cultivars to evaluate the effect of mutation in each species. The results from this study can help to discriminate various cultivars, which could be further processed for dietary supplements [13].

Plants from Bombacoideae are known for their medicinal properties. Das et al. reported systematic reviews to provide information of taxonomy, phytochemistry, biological activities, and application in food from the subfamily of Bombacoideae [14].

Metabolomics is comprehensive study of metabolites in organisms including plants. This approach can provide useful information related to controlling growth and the development of plants. Patel et al. reported a review article that described recent analytical instrument and techniques applied for studying metabolites in plants. Additionally, bioinformatics tools and metabolomics databases for plants are mentioned in this article. Collectively, this review article provides overall workflow and information for plants metabolomics studies [15].

Overall, the 15 contributions to this Special Issue “Isolation and Analysis of Characteristic Compounds from Herbal and Plant Extracts” discuss the biochemical activities of plant extracts and characterized compounds related to activities. These types of research studies ensure the development of new bioactive compounds that could be used as potential agents for the treatment of diseases in years to come.

## Data Availability

All data included in the main text.
